# Bi-allelic variants in *BRF2* are associated with perinatal death and craniofacial anomalies

**DOI:** 10.1186/s13073-025-01463-3

**Published:** 2025-04-14

**Authors:** Francesca Mattioli, Rún Friðriksdóttir, Anne Hebert, Sissy Bassani, Nazia Ibrahim, Shagufta Naz, Jacqueline Chrast, Clara Pailler-Pradeau, Ásmundur Oddsson, Patrick Sulem, Gisli H. Halldorsson, Páll Melsted, Daníel F. Guðbjartsson, Flavia Palombo, Tommaso Pippucci, Nayereh Nouri, Marco Seri, Emily G. Farrow, Carol J. Saunders, Nicolas Guex, Muhammad Ansar, Kari Stefansson, Alexandre Reymond

**Affiliations:** 1 Center for Integrative Genomics, University of Lausanne, Genopode Building, CH-1015 Lausanne, Switzerland; 2 deCODE Genetics/Amgen Inc, Reykjavik, Iceland; 3 Lahore College for Women University, Lahore, Pakistan; 4 School of Engineering and Natural Sciences, University of Iceland, Reykjavik, Iceland; 5 IRCCS Istituto Delle Scienze Neurologiche, Programma Di Neurogenetica, Bologna, Italy; 6 IRCCS Azienda Ospedaliero-Universitaria Di Bologna, Bologna, Italy; 7 Craniofacial and Cleft Research Center, Isfahan University of Medical Sciences, Isfahan, Iran; 8 University of Missouri-Kansas City School of Medicine, Kansas City, MO USA; 9 Department of Pathology and Laboratory Medicine, Children’s Mercy, Kansas City, MO USA; 10 Bioinformatics Competence Center, University of Lausanne, Lausanne, Switzerland; 11 Department of Ophthalmology, University of Lausanne, Jules-Gonin Eye Hospital, Fondation Asile Des Aveugles, Lausanne, Switzerland; 12 Faculty of Medicine, School of Health Sciences, University of Iceland, Reykjavik, Iceland; 13 Health2030 Genome Center, Foundation Campus Biotech Geneva, Geneva, Switzerland

**Keywords:** Autosomal recessive, RNA polymerase III, Craniofacial anomalies, BRF2

## Abstract

**Background:**

Variants in genes encoding multiple subunits of the RNA Polymerase III complex which synthesizes rRNAs, tRNAs, and other small RNAs were previously associated with neurological disorders, such as syndromic hypomyelination leukodystrophies, pontocerebellar hypoplasia, and cerebellofaciodental syndrome. One new such candidate is *BRF2*, which encodes a TFIIB-like factor that recruits the RNA polymerase III complex to type 3 promoters to initiate transcription of U6, RnaseP, and 7SK RNAs.

**Methods:**

We combined sequencing with functional analyses to investigate the effects of *BRF2* variants.

**Results:**

We observe that a previously reported significant underrepresentation of double transmission of a splice variant results in recessive lethality in three large Icelandic families with multiple perinatal losses. Using data aggregation, we identified an additional seven individuals worldwide from three unrelated families carrying biallelic variants in *BRF2*. Affected individuals present a variable phenotype ranging from severe craniofacial anomalies with early death to intellectual disability with motor and speech development. In silico 3D modelling and functional analyses showed functional impairment of the identified variants, e.g., differences in target loci occupancy. Zebrafish knocked down for the orthologous *brf2* presented with abnormal escape response, reduced swimming velocity and head size, and craniofacial malformations. These defects were complemented by the human wild-type but not mutated BRF2 mRNA further demonstrating their deleteriousness.

**Conclusions:**

Overall, our results support the association of biallelic *BRF2* variants with a novel neurodevelopmental disease and provide an additional link between RNA polymerase III, its targets and craniofacial anomalies.

**Supplementary Information:**

The online version contains supplementary material available at 10.1186/s13073-025-01463-3.

## Background

Congenital disorders are heterogeneous conditions characterized by morphological, biochemical, or functional defects that appear during the intrauterine life, at birth, or postnatally (World Health Organization: https://www.who.int/health-topics/congenital-anomalies [1]) and could lead to life-long disability or even early death [2, 3]. They are a major health issue as about 6% of babies worldwide are born with such conditions without accounting for spontaneous abortions and stillbirths, congenital anomalies are a major health issue. Many of these conditions have a genetic origin that could be ascribed to a single-gene defects [4, 5]. While the leading cause in Western countries are autosomal dominant de novo variants [6–8], autosomal recessive inheritance is common in countries with frequent parental consanguinity as offspring of consanguineous parents are homozygous for a considerable fraction of their genome [9–11]. Accordingly, consanguineous populations such as Pakistan with 63% and Iran with 40% of intrafamilial marriages have a high prevalence of birth defects [12, 13]. While next-generation sequencing techniques allowed identifying thousands of genes linked to Mendelian conditions [14], about half of the cases remain without genetic diagnosis [4, 6, 7]. This suggest that many causative genes are still unknown in particular recessive ones and that consanguineous families provide a unique opportunity to identify novel recessive causative genes [15].

Large-scale sequencing studies reported genes with a significant paucity of biallelic predicted loss-of-function (pLoF) variants [16–20], providing valuable insights into the genetic of recessive lethality. Whole-genome sequencing and variants imputation of 1.52 million individuals from Iceland, Denmark, Norway, Sweden, Finland, and the UK identified 25 genes with a significant underrepresentation of double transmissions of pLoF variants from heterozygous parents to their offspring. For example, a deficit of homozygous genotypes has been reported for a splice donor variant c.214 + 1G > A at the end of exon 2 of *BRF2* (MAF = 0.83%, expected homozygotes = 11.8 vs. observed homozygotes = 0) [16, 17]. This donor splice variant is nearly absent in public databases (MAF in gnomAD v4 = 0.0009294%) consistent with a founder effect in Iceland.

BRF2 (TFIIB-related factor 2) is an RNA polymerase III transcription initiation factor subunit that shares the N-terminal zinc ribbon and core domain with TFIIB and BRF1 [21, 22]. BRF2 is only present in vertebrate and its C-terminal is unique and essential for binding type 3 promoters [23, 24]. Examples include the U6 small nuclear RNA, MRP RNAs, 7SK RNA, Y RNAs, ribonuclease P, and selenocysteine-tRNA [25], thus implicating BRF2 in several processes such as RNA splicing, mitochondrial RNA processing, RNA polymerase II transcriptional elongation, tRNA processing, and oxidative stress [24, 26]. Variants in several subunits of the RNA polymerase III complex have been linked to neurological and/or cognitive disorders (e.g., *POLR3A:* MIM#607,694, 264,090 [27, 28]; *POLR3B:* MIM#614,381 [29, 30]; *POLR3K*: MIM#619,310 [31]; *POLR3GL*: MIM#619,234 [32, 33]; *POLR1C* MIM#616,494, 248,390 [34, 35]). Bi-allelic variants in *BRF1*, which guides RNA polymerase III to type 1 and type 2 promoters [36], were associated with cerebellofacialdental syndrome (CFDS; MIM#616202) [37–41].

We report six families with bi-allelic variants in *BRF2* presenting with early mortality, brain, and craniofacial anomalies and/or neurodevelopmental disorders (NDD). In silico 3D modelling, transcriptome profiling, cellular assays, and ChIP chromatin immunoprecipitation (ChIP) suggest functional impairment of the identified variants*.* Zebrafish ablated for the *BRF2* ortholog showed morphological and neurological deficits that could be complemented by human *BRF2*, but not by isoforms carrying the variants identified in affected family members.

## Methods

### Samples

#### Family 1, 2, 3

The methods used for whole-genome sequencing (WGS) were as follows: paired-end libraries for sequencing were prepared from DNA samples (derived from blood or buccal swabs) using Illumina preparation kits (TruSeq DNA, TruSeq Nano, or TruSeq PCR-Free) according to the manufacturer’s instructions. Paired-end sequencing-by-synthesis (SBS) was performed on Illumina sequencers (GAIIx, HiSeq 2000/2500, HiSeq X, or NovaSeq) to a target depth of 30X . Read lengths varied from 2 × 76 bp to 2 × 150 bp, depending on the instrument and/or sequencing kit used. Reads were aligned to the human genome assembly GRCh38 using the Burrows–Wheeler Aligner (BWA) version 0.7.10 [42]. Alignments were merged into a single BAM file and marked for duplicates using Picard 1.117. Only non-duplicate reads were used for the downstream analyses. Variants were called using version 2014.4–2-g9ad6aa8 of the Genome Analysis Toolkit (GATK) [43], reads were called with GATK using a multi-sample configuration. The effect of sequence variants was annotated using release 80 of the Variant Effect Predictor (VEP-Ensembl). To be able to filter out genotypes over a certain frequency threshold, we used allelic frequencies from phased genotypes of 32.5 million SNPs and INDELs from 28,075 Icelanders who have been whole-genome sequenced at deCODE genetics [44]. The offsprings of families 1–3 were born in the 1950s, 1970s, and 1920s, respectively; hence, samples from the deceased offsprings were not available.

#### Family 4

Genomic DNA was extracted from peripheral blood samples collected in EDTA anticoagulant with the GenElute Blood Genomic DNA Kit (Sigma Aldrich, Missouri, USA) and with the QIAamp DNA Blood Mini (Qiagen, Venlo, Netherlands), following the manufacturer’s instruction. Targeted capture and enrichment were performed using the Nextera Rapid CaptureExome kit (Illumina Inc., San Diego, CA), and libraries were sequenced as 100-bp paired-end reads on Illumina HiSeq2000 platforms (Illumina). Generated reads were treated following a general pipeline described elsewhere [45]. The mean bait coverage was on average 62-fold with an average 81% of the bases being covered at least 20-fold. H3M2 [46] was used for the identification of runs of homozygosity (ROHs) from WES alignments. Variants were analyzed through an autozygosity-driven approach including detection of long ROHs, likely autozygous (ROHs > 1.5 Mb), and prioritization of candidate variants as described [45]. Segregation analysis of candidate variants was performed by Sanger sequencing. The identified variants from this analysis are reported in Table 4 (Family GME_25) in [45].

#### Family 5

Genomic DNA was extracted from peripheral blood and sequenced in the Genomic Medicine Center at Children’s Mercy Hospital as previously described [47]. Briefly, the samples were prepared for sequencing using the Illumina TruSeq PCR Free library preparation kit and sequenced with 2 × 125 base pair paired-end sequencing reads on an Illumina HiSeq 4000 (Illumina Sequencing, San Diego, CA, USA) to a mean depth of 30x. All analyses were completed on GRCh38. Dragen v3.6.3 (Illumina, San Diego, CA) was used for alignment and variant calling. Variant annotation was completed using custom software RUNES, as previously described [48].

#### Family 6

Genomic DNA was isolated from whole blood and sent for sample preparation, library construction, sequencing, and data processing to the Lausanne Genomic Technologies Facility. In brief, samples were enriched for exonic DNA using the xGen Exome Research Panel v2 (Integrated DNA Technologies, Coralville, IA, USA) and sequenced with 2 × 150 base pair paired-end sequencing reads on an Illumina HiSeq4000 (Illumina Sequencing, San Diego, CA, USA). The mean bait coverage was on average 155-, 107-, and 146-fold with on average 97%, 95%, and 97% of the bases being covered at least 20-fold, respectively for individual II.1, II.2, and II.3. Sequencing data were processed by an automated pipeline, as described previously [49, 50]. Briefly, variants shared between the three affected siblings were retrieved and filtered using the Varapp software. Variants passing the quality filter and predicted to have an impact on protein function were retained, before filtering according to all possible inheritance patterns with a minor allele frequency (MAF) below 1% in different population databases. Subsequent variant prioritization used a combination of pathogenicity prediction scores and literature search. In addition, whole exome sequencing data of each sibling was analyzed separately for candidate variants in known intellectual disability genes (PanelApp version 3.2) [51]. Segregation analysis of candidate variants was performed by Sanger sequencing. Whole-exome sequencing analysis of three of the four affected individuals (II:1–3) identified six homozygous candidate variants. Five of them were excluded either because of their low pathogenicity scores or as the function of the encoded protein does not correlate with the patients’ phenotype (Additional file 1: Table S1). Two sets of possibly compound heterozygous variants were identified in gene previously associated with ID (intellectual disability) [(NM_001252100.1(*KIF21B*):c.3653C > T p.(Thr1218Met) and c.4450G > A p.(Glu1484Lys); NM_012090.5(*MACF1*):c.10619C > T p.(Ala3540Val) and c.15089G > A p.(Arg5030Gln)] but they were subsequently excluded as segregation analysis by Sanger sequencing showed that all four variants mapped on the maternal allele and did not segregate with the disorder.

### In silico models of missense variants in *BRF2*

BRF1 and BRF2 3D protein models were created using the Swiss-Pdb Viewer [52]. The Protein Data Bank (PDB) entry 5N9G (human TFIIIB-TBP/Brf2/DNA and SANT domain of Bdp1) was used as a template to superpose the other human BRF2 crystal structures available, bound to various response elements (entries 4ROE Human TFIIB-related factor 2 (Brf2) and TBP bound to RPPH1 promoter, 4ROD bound to TRNAU1 promoter, 4ROC bound to U6#2 promoter). Structures were superposed using the iterative magic fit option of Swiss-PdbViewer [53]. Superposition was great (Carbon Alpha rmsd onto 5N9G of 1.07 Angstrom for 447 residues, 1.19A for 458 residues 1.17A for 460 residues, respectively for the three structures), demonstrating an identical binding to all response elements. Since the N-terminal part of BRF2 that includes Gly11 was not resolved, the AlphaFold model Q9HAW0 and chain A of the nuclear magnetic resonance structure 1DL6, identified with HHPRED, were utilized to predict its relative position to the crystallized BRF2 structure and DNA . [54–56]. Additionally, the relative position of BRF2 to RNA polymerase III was predicted using PDB entry 6F40 (*Saccharomyces cerevisiae*). BRF1 Alphafold model AF-Q92994-F1-model_v2.pdb was superposed on chain V of pdb entry 6F40 retaining the best superposition of the “Explore Fragments Alternate Fits” option of SwissPdbViewer [53].

### In vitro assays

#### *BRF2* cloning and site-directed mutagenesis

*BRF2* clones were created through conventional cloning techniques using 5-alpha competent *E. coli* bacteria (New England Biolabs, Ipswich, MA, USA) and verified by sequencing at Eurofins Genomics (Ebersberg, Germany) with the following primers: Forward 5′-ATGTCTAGACCAGGCAGAGGC-3′ or 5′-CTGCTTGTGTGTTGGAGGTC-3′; Reverse 5′-CGTCGCCGTCCAGCTCGACCAG-3′. *BRF2* cDNA (NM_018310.4, lacking start and stop codon) was amplified from a pCITE-Brf2 plasmid (a kind gift from Prof. N. Hernandez) with the Phusion High-Fidelity DNA Polymerase (New England Biolabs, Ipswich, MA, USA) and the following primers: Forward 5′-ATGTCTAGACCAGGCAGAGGC-3′; Reverse 5′-TCCGGGAGGGTTAGGGACACT-3′. The amplified product was then inserted into a pCR8/GW/TOPO entry vector, following the manufacturer’s protocol (pCR8/GW/TOPO TA Cloning Kit; Invitrogen, Thermo Fisher Scientific Inc., Waltham, MA, USA). Using the Gateway LR Clonase II enzyme mix (Invitrogen, Thermo Fisher Scientific Inc., Waltham, MA, USA), the *BRF2* insert was transferred into the destination vector pDEST-CMV-3xFLAG-GW-EGFP (#122,845 [57]; Addgene, Watertown, MA, USA) and pCSF107mT-GATEWAY-3’-FLAG (#67,619; gift from Todd Stukenberg, Addgene, Watertown, MA, USA). The mutant pDEST-CMV-3xFLAG-BRF2-EGFP and pCSF107mT-BRF2-3’-FLAG expression plasmids were then generated by site-directed mutagenesis, using specific, complementary primer pairs for each mutation and Pfu DNA polymerase (Promega, Fitchburg, WI, USA). After PCR amplification following the manufacturer’s protocol, the template DNA was digested by the DpnI restriction enzyme (New England Biolabs, Ipswich, MA, USA). Each plasmid was then verified by Sanger sequencing.

#### Western blot

2 × 10^5^ HEK293T cells were plated in 12-well plates with Dulbecco’s modified Eagle’s medium (DMEM; Gibco, Thermo Fisher Scientific, Waltham, MA, USA) supplemented with 10% fetal bovine serum (FBS; Sigma-Aldrich, Merck KgaA, Darmstadt, Germany) the day before the transfection. Using a 3:1 ratio, 1.1 µg DNA was transfected with FuGene Transfection Reagent (Promega, Madison, WI, USA) in a final volume of 50 µl with Opti-MEM (Gibco, Thermo Fisher Scientific, Waltham, MA, USA). For protein extraction, cells were harvested 20 h after transfection using radioimmunoprecipitation assay (RIPA) buffer, which consisted of 50 mM Tris–HCl pH 7.5, 150 mM NaCl, 0.25% Na-deoxycholate, 1% NP40 and water, supplemented with fresh 1 × proteinase-phosphatase inhibitor cocktail (Thermo Fisher Scientific Inc., Waltham, MA, USA). Harvested cells were incubated for 1 h on a shaker at 4 °C, followed by 2 h at − 80 °C and thawing on ice with intermittent vortexing. Lastly, samples were spun down for 20 min at 4 °C and 13,000 rpm. Protein concentrations were determined using the Pierce BCA protein assay kit (Thermo Fisher Scientific Inc., Waltham, MA, USA). To assess BRF2 expression, 5 µg of protein was loaded on 10% Mini-PROTEAN TGX precast polyacrylamide gels (Bio-Rad, Hercules, CA, USA) and transferred onto PVDF membranes. Protein-binding sites were blocked by incubating the membrane in 5% milk powder in PBS-T solution (1 × PBS and 0.1% Tween20) for 1 h at room temperature. The primary, monoclonal antibodies (mouse anti-FLAG [F1804; Sigma-Aldrich, Merck KgaA, Darmstadt, Germany] and rabbit anti-β-actin [A2066; Sigma-Aldrich, Merck KgaA, Darmstadt, Germany]) were diluted 1:2000 and 1:5000, respectively, in blocking solution and incubated overnight at 4 °C on a shaker. After washing in PBS, the secondary antibodies (goat anti-mouse HRP [W402B; Promega, Madison, WI, USA] and goat anti-rabbit IgG-HRP [sc-2030; Santa Cruz Biotechnology, Dallas, TX, USA]) were diluted 1:2500 and 1:5000, respectively, in blocking solution and incubated for 1 h at room temperature. After washing in PBS, protein bands were visualized using the Pierce ECL Western Blotting substrate (Thermo Fisher Scientific Inc., Waltham, MA, USA) on a fusion FX6 edge imaging system (Vilber Lourmat, Collégien, France). Western Blotting was performed in biological triplicates.

#### Immunofluorescence imaging

For immunostaining, transfected HEK293T cells were fixed in 4% PFA in 1 × PBS for 10 min at room temperature. After washing in 1 × PBS, cells were incubated with saturation solution (10% FBS, 1% BSA, 0.2% Triton X-100, and 1 × PBS). The primary monoclonal antibody (mouse anti-FLAG [F1804; Sigma-Aldrich, Merck KgaA, Darmstadt, Germany]) was diluted 1:500 in the saturation solution and incubated overnight at 4 °C on a shaker. After washing in PBS, the secondary antibody (goat anti-mouse AlexaFluor568 [A11019; Invitrogen, Thermo Fisher Scientific Inc., Waltham, MA, USA]) was diluted 1:1000 in saturation solution without Triton X-100 for 1 h at room temperature. After washing in PBS, cells were mounted onto microscopic slides with Vectashield and incubated overnight at 4 °C. Alternatively, DAPI was diluted 1:10,000 and incubated for 10 min at room temperature. After washing in PBS, cells were mounted onto microscopic slides with Mowiol 4–88 and incubated overnight at 4 °C. Images were acquired with a ZEISS LSM 880 confocal microscope with Airyscan (Carl Zeiss AG, Oberkochen, Germany) using the Plan-Apochromat 63x/1.4 oil DIC M27 objective and the ZEN software (RRID:SCR_013672). Z-stacks were obtained at 405 nm, 488 nm, and 594 nm. Images were analyzed with the Z Project function in Fiji-ImageJ [58]. Immunostaining was performed in five biological replicates.

### Chromatin immunoprecipitation

Nuclei preparation was performed as described in [59]. Briefly, 10 million Lipofectamine 3000-transfected (L3000001, Invitrogen) HEK293T cells were harvested, washed with PBS, crosslinked for 10 min in 333 mM formaldehyde, quenched in 250 mM Tris pH 8.0, rinsed with PBS, and freezed at − 80 °C. Fixed cells were resuspended and dissociated for 10 min at 4 °C on a rotating wheel in 50 mM HEPES–KOH pH 7.4 140 mM NaCl 1 mM EDTA 0.5 mM EGTA 10% Glycerol 0.5% NP40 0.25% Triton X-100 and protease inhibitor cocktail tablet (11,836,153,001, Roche cOmplete Mini), washed thrice in 10 mM Tris pH 8.0 200 mM NaCl 1 mM EDTA 0.5 mM EGTA and protease inhibitor cocktail tablet, before nuclei sonication in 10 mM Tris pH 8.0 1 mM EDTA 0.15% SDS and protease inhibitor cocktail tablet on a Covaris S2 AFA focused ultra-sonicator (Covaris, Woburn, MA, USA) for 20 min at 5% duty cycle (140W, 200 cycles). Sonicated lysates were resuspended to a final concentration of 1% Triton X-100 and 150 mM NaCl and mixed with 10 µl of packed 50 mM Tris–HCl pH 7.5 150 mM NaCl-prewashed anti-FLAG M2 magnetic beads (Millipore, M8823). After incubation for 3 h at room temperature under rotation, the beads/lysates mixes were subjected to a magnetic field to discard the supernatant before washing the beads three times with 50 mM Tris–HCl pH 7.5 150 mM NaCl. To elute FLAG-tagged proteins, the magnetic beads were incubated for 30 min at 4 °C under rotation in 50 µl 50 mM Tris–HCl pH7.5 150 mM NaCl containing 150 ng/µl 3xFLAG peptide (Sigma-Aldrich, F4799) and subjected to a magnetic field to recover the supernatant. After repeating this elution steps a second time, the 100 µl of the two combined recovered supernatants were first incubated for 1 h at 37 °C after addition of 1 µl of RNAse A 20 µg/µl, before reversing the crosslink by addition of 2 µL of proteinase K at 20 µg/µL and incubation at 65 °C overnight with agitation. Immunoprecipitated DNA was recovered from decrosslinked and RNAse-treated supernatants using MinElute PCR purification kit (Qiagen). Occupancy of the *RMRP*, *RNU6-2*, and *Selenocysteine-tRNA* BRF2-bound loci was then assessed by quantitative PCR using GoTaq qPCR Master Mix (Promega, A6001) and the following pairs of primers (*RMRP*: 5′-CGCCACCAACTTTCTCACCCTAA-3′ and 5′-ATACAGGCCTTCAGCACGAACC-3′; *RNU6-2*: 5′-TTCTGCAACATACCACTGTAGGA-3′ and 5′-TATATGTGCTGCCGAAGCGA-3′; *SeCys*: 5′-TCAGTGGTCTGGGGTGCAGG-3′ and 5′-GTCCGGTTCGATAAGTAAGATTTAAGGC-3′) in a QuantStudio™ 6 Flex Real-Time PCR System (Applied Biosystems). Amplification of a control locus, *EEF1A1*, a non BRF2-bound locus, with primers 5′-CTGAACCATCCAGGCCAAAT-3′ and 5′-GCCGTGTGGCAATCCAAT-3′ was used to normalize the quantity of input DNA between samples.

### RNA expression of the splicing *BRF2* variant

Blood samples of 323 Icelandic individuals heterozygous for the *BRF2* (NM_018310.4):c.214 + 1G > A variant and 17,351 Icelandic individuals without the variant were used to assess BRF2 expression in blood. The adipose RNA-seq dataset comprised 18 individuals heterozygous for the *BRF2* (NM_018310.4):c.214 + 1G > A variant, while 752 individuals were negative. Fragment counts were determined by alignments to *BRF2* exon 1, considering splicing to exon 2, and alternatively counting alignments skipping exon 2 while splicing to exon 3. Given the homology between the terminal ends of exon 1 and exon 2, only fragments with a minimum 5-base pair overhang were included. The percentage spliced in (PSI) was calculated following the methodology outlined in LeafCutter [60].

### In vivo characterization of *brf2* KD zebrafish

#### Zebrafish husbandry and ethical statement

Zebrafish (*Danio rerio*) of the AB strain were maintained at 28.5 °C in a 12/12-h light/dark cycle. Adult zebrafish were housed in an Active Blue rack recirculating system (Tecniplast S.p.A., Buguggiate, Italy) with maximally 20 fish per tank. Zebrafish embryos and larvae were kept in petri dishes. Their aquaculture was replaced regularly, and the death rate was monitored. This research was in compliance with the European Convention for the Protection of Vertebrate Animals used for Experimental and other Scientific Purposes (ETS number 123) and followed the Guide for the Care and Use of Laboratory Animals by the National Research Council [61]. The housing conditions were approved the Vaud cantonal authority (VD-H21). Larvae were anesthetized with tricaine (MS-222) at 5 days post fertilization (dpf).

#### Zebrafish CRISPR-Cas9 microinjection

Three synthetic single-guide RNAs (sgRNAs) targeting *brf2*, the zebrafish BRF2 ortholog (Thermo Fisher Scientific Inc., Waltham, MA, USA) and corresponding primer pairs (Thermo Fisher Scientific Inc. or Sigma-Aldrich, Merck KGaA, Darmstadt, Germany) were designed with the CHOPCHOP, SnapGene and Primer3 softwares as well as the in silico PCR tool of the University of California in Santa Cruz genome browser (Additional file 1: Table S2) [62–64].

Random crossings of adult and sexually mature zebrafish were set up in breeding tanks, where two females were separated from three males using a see-through separator. Removal of the separator the following morning led to natural spawning. Viable eggs were collected for the generation of F0 KD zebrafish, 1 nl from a mix containing 100 ng/µl of each of the three sgRNAs, 200 ng/µl Cas9 (Life Technologies Europe BV, Thermo Fisher Scientific Inc., Waltham, MA, USA), 200 mM KCl and PhenolRed was microinjected into one-cell stage embryos. In mock-injected larvae, the Cas9 enzyme was replaced by the same amount of water. Wildtype and mutated human *BRF2* mRNA—obtained from the in vitro transcription (mMESSAGE mMACHINE SP6 Transcription kit, Invitrogen, Thermo Fisher, Waltham, Massachusetts, USA) of the pCSF107mT-BRF2-3’-FLAG—were co-injected for a final amount of 200 pg or 100 pg according to the experiment. Injection of 200 pg of human *BRF2* WT or mutated mRNA alone with 200 mM KCl did not result in toxicity.

#### Characterization of CRISPR-Cas9 editing events

DNA of individual zebrafish larvae was extracted at 5 dpf using a mix of 10 mM Tris HCl pH 8.8, 2 mM EDTA pH 8, 0.2% Triton X-100, and water in a final volume of 14.85 µl per zebrafish larvae. After freshly adding 0.15 µl proteinase K to each sample, tubes were incubated for 1 h at 55 °C, followed by 10 min at 98 °C. The QIAprep&amp CRISPR kit and respective primer pairs (Qiagen Hilden, Germany; Additional file 1: Table S2) were used to amplify the target region of each sgRNA. PCR amplification was confirmed on a 1% agarose gel with GelRed™ Nucleic Acid Gel Stain (Biotium, Fremont, CA, USA) and a DNA ladder (BenchTop, Promega, Fitchburg, WI, USA). To determine the CRISPR-Cas9 targeting efficiency of each sgRNA, a cleavage assay using the T7 endonuclease I was performed according to the manufacturer’s protocols (New England Biolabs, Ipswich, MA, USA). After stopping the enzyme reaction, the zygosity of the zebrafish larvae was determined on a 2% agarose gel with GelRed™ Nucleic Acid Gel Stain (Biotium) and a 100-bp ladder (BenchTop, Promega, Fitchburg, WI, United States). The efficiency of each sgRNA was assessed from ten *brf2*-KD larvae.

#### Touch-response test

At 3 dpf touch-response tests were performed in the morning. Zebrafish larvae were placed individually in the center of a 10-cm Petri dish, touched with a thin tip gently at the tail, and their escape response was classified into the following categories: normal swimming, short and/or slow swimming, looping swimming, pinwheel swimming, startle response, no response, no response due to malformations. Results of four independent experiments were pooled.

#### Locomotion assay

At 5 dpf zebrafish larvae were placed in individual wells of 96-well plates filled with 200 µl water. Plates were placed inside an automated video-tracking device (ZebraBox™, Viewpoint, Lyon, France). Larval locomotion was tracked using the ZebraLab software (Viewpoint, Lyon, France) with the transparent background mode. Movements greater than 6 mm/s were considered fast, and a larva was considered inactive for movements between 6 and 3 mm/s. After a 15-min adaptation phase in the light, the velocity of the fish was tracked in a 15-min dark phase. In the analysis, the net and global velocity was calculated for each condition. Results of multiple independent experiments were pooled.

#### Morphological analysis

At 5 dpf, images of the zebrafish were taken for morphological assessment and quantifying the head width and size. Images were acquired with a LEICA M165 FC microscope using a × 10 objective and analyzed with the set scale and measure functions in Fiji-ImageJ [58]. Measurements were obtained from three independent experiments.

#### Alcian blue staining

The day after the injection, zebrafish larvae were treated with 0.06% 1-phenyl 2-thiourea (PTU) to block pigmentation. At 5 dpf, they were euthanized with 0.0168% tricaine (MS-222, Sigma-Aldrich) and fixed overnight at room temperature with paraformaldehyde (PFA) at 4%. After three washes with 1X PBS and 0.1% Tween-20, specimens were bleached for 2 h at room temperature with 30% hydrogen peroxide and stained with Alcian Blue solution (1% concentrated hydrochloric acid, 70% ethanol, 0.1% Alcian blue [Sigma-Aldrich]) at 4 °C overnight. They were then rinsed three times and incubated for 20 min at room temperature with acidic ethanol (5% concentrated hydrochloric acid, 70% ethanol). Zebrafish larvae were then rehydrated with graded series of acidic ethanol of 10 min each (1st: 75% acidic ethanol; 2nd: 50% acidic ethanol; 3rd: 25% acidic ethanol; 4th: water 0% ethanol) and submerged in a 50% glycerol-1% KOH solution. Ventral view pictures of the stained zebrafish were acquired with the LEICA M165 FC microscope using a × 10 objective. Results of multiple independent experiments were pooled.

#### Statistical analyses

All statistical analyses were performed in GraphPad Prism 9.3.1. Swimming velocity, head width, and size were analyzed by Kruskal–Wallis tests, followed by Dunn post hoc tests in case of statistically significant H-statistics. Χ^2^ test was used to assess statistical significance among the evaluated conditions for malformed cartilaginous structure. A *p*-value below 0.05 was considered statistically significant.

## Results

### Biallelic* BRF2* variants and phenotypes

**Fig. 1:**
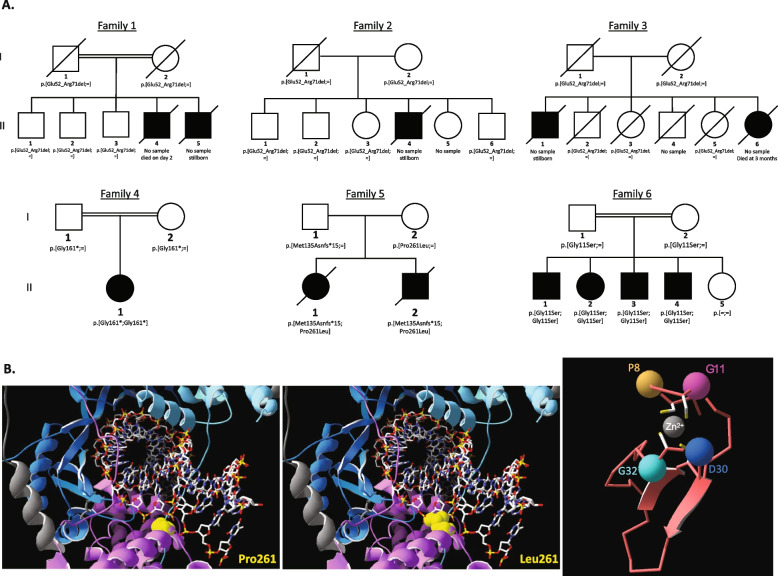
Identified biallelic variants in *BRF2*.** A** Pedigrees and the genotypes of the reported families. The offsprings of Icelandic families 1–3 were born in the 1970s, the 1950s, and 1920s, respectively. **B** 3D protein modelling of the identified missense variants. Pro261 highlighted in yellow is in close vicinity with the DNA backbone between two phosphate groups (left panel); the Pro261 is replaced by a Leucine highlighted in yellow in the middle panel. The bulkier sidechain of Leu261 will likely collide with the DNA. The right panel presents a schematic representation of the zinc-finger highlighting the conserved residues Gly11 (purple), Asp30 (dark blue), Gly32 (light blue), and Pro8 (gold). Coordination of the zinc-atom (gray) by the four conserved Cysteine (shown in sidechains) will expose the sidechains of Pro8, Gly11, Asp30, and Gly32

In our population set of 170,000 array-genotyped Icelandic individuals imputed from genome sequencing of 73,000 Icelanders, we identified 28 couples where both individuals are heterozygous for the previously reported splicing donor variant *BRF2* (NM_018310.4):c.214 + 1G > A (CADD = 34; spliceAI = 1; MaxEntScanDiff = 8.182) [65–67]. The genotype is available for 29 out of 66 offspring of these couples: 22 are heterozygous (76%), 7 are homozygous wild type (24%), and none are homozygous mutant. Couples heterozygous for the *BRF2* splice variant did not have a higher risk of miscarriage compared to non-carrier couples matched on year of birth (OR = 2.14 [95%CI 0.82, 5.55], *P* = 0.094, N carrier couples = 21, N miscarriage = 10). Data of early mortality is available for three large families where both parents are heterozygous for this variant and experienced perinatal loss of one or more children (totally 5 of 17 offspring among 3 families; Families 1–3; Fig. 1A). Of the 12 surviving offspring, 10 were available for genotyping and were all heterozygous. Although the deceased individuals were unable to be genotyped, these families provide convincing evidence of *BRF2* as a candidate gene.

Through exome sequencing and data aggregation via GeneMatcher [68], we identified three additional families with bi-allelic variants in *BRF2*. The proband of one of these families (Family 4—II:1; Fig. 1A) was born from consanguineous Iranian parents. She was diagnosed with Treacher-Collins syndrome, a rare congenital disorder characterized by severe craniofacial anomalies [69]. She presented with hearing impairment, soft cleft palate, microcephaly, and facial dysmorphism (preauricular left ear tag, medially flared eyebrows, telecanthus, left epicanthus, down slanting palpebral fissures, curved eyelashes, malar hypoplasia, long nose with convex profile, low inserted columella and underdeveloped alae, short deep philtrum, thick everted vermilion of the lower lip). The size of the feet was smaller than that of the hands. This proband was homozygous for a stop-gain variant in *BRF2* (NM_018310.4):c.481G > T; p.(Gly161*) [45]. This variant has a CADD score of 36 [70] and is not observed in gnomAD v4.0.0 (https://gnomad.broadinstitute.org/) [71], REGENERON [72], Iranome [73], and TopMed Freeze5 (https://bravo.sph.umich.edu/freeze5/hg38/).

The fifth family comprised two affected children from unrelated parents (Family 5—II:1–2; Fig. 1A). The first child was born at 37 weeks of gestation, following a pregnancy complicated by polyhydramnios and bowel obstruction. She was suggested to suffer from a new form of Baller-Gerold syndrome (MIM#218600) [74], exhibiting, at birth, coronal synostosis, microcephaly, hypertelorism, a small beaked, nose, retrognathia, shortened right radius, and absent left radius, with radial deviation of the hands and contractures of all fingers. She was found to have anemia, leukocytosis, marked eosinophilia, and thrombocytopenia, and developed a significant rash by 1 month of age. She had recurrent infections and died at ~ 2 months of age due to bacterial infection. Her brother presented with frontal bone hypoplasia with bilateral coronal synostosis, micrognathia, small orbits, low-set ears, downward slanting palpebral fissures, and significantly decreased B-cell CD19 subsets. He subsequently developed ichthyosiform erythroderma and eosinophilic myeloid hyperplasia. At 2 years of age, he received a bone marrow transplant, complicated by graft versus host disease. He had developmental and speech delays. He died at 3 years of age due to complications of graft versus host disease and infection. These siblings were compound heterozygous for variants in *BRF2*(NM_018310.4):c.782C > T ; p.(Pro261Leu) inherited from their mother, and (NM_018310.4):c.404_409delinsA; p.(Met135Asnfs*15) inherited from their father. The frameshift variant introduces a stop codon, while the missense variant affects a highly conserved residue (Additional file 1: Figure S1). Multiple predictors support a deleterious effect of p.(Pro261Leu) on BRF2 function (PolyPhen2 = 0.977; SIFT = 0.01, CADD = 25.4; REVEL = 0.631; GERP = 5.6399; PhastCons = 1) [75–77]. While the frameshift variant is absent from gnomAD v4.0.0, TopMED and REGENERON, the missense variant is present at a MAFs equal to 0.001673%, 0.0024%, and 0.0007% in these three databases, respectively. We evaluated the effect of the missense variant by in silico 3D modelling. The mutated Pro261 residue is found in the C-terminal part of the BRF2 C-cyclin repeat [24], in contact with the template strand DNA backbone at positions −2 and −3 relative to the TATA (Fig. 1B, Additional file 1: Figure S2A). The preceding amino acid, Tyr260, is in direct contact with the template strand nucleotide C'−4 of the TATA (pdb: 4ROC) [24]. This suggests that the conformation of this BRF2 region is likely important for interaction with the DNA. Consistent with this hypothesis, mutating nucleotides at positions −3 and −4 of the TATA box was shown to affect the complex formation [24]. Further supporting the deleterious impact of modifying this codon, our modelling showed that BRF2 Pro261 structurally overlaps with the BRF1 Thr259 residue, whose variant p.(Thr259Met) is associated with CFDS and leads to decreased promoter occupancy at tRNA and U6 snRNA loci [37] (Additional file 1: Figure S2B). All BRF1 residues found mutated in CFDS map to either the N- or the C-cyclin repeats and possibly influence DNA binding [37–41] (Additional file 1: Figure S2B).

The sixth family was identified within a cohort of Pakistani families assessed for intellectual disability (ID). Four affected siblings (Family 6 – II:1–4; Fig. 1A) presented with moderate ID, and delays in motor and speech development. Proband II:2 (16-year-old female) and II:3 (14-year-old male) additionally reported with mild hearing and vision impairment, as well as feeding difficulties. Malnutrition during pregnancy and severe jaundice as a neonate was also noted for proband II:3. No additional clinical characteristics were described for proband II:1 (18-year-old male) and II:4 (6-year-old male). Exome sequencing of three of the four affected individuals (II:1–3) identified a homozygous missense variant in *BRF2* (NM_018310.4):c.31G > A ; p.(Gly11Ser) that segregates with the phenotype in the four affected siblings, is heterozygous in both parents, and is not present in the healthy sister (Family 6; Fig. 1A). While this variant is not reported in Iranome, REGENERON or in our local database of 400 Pakistani individuals, it was identified in TopMED with (MAF of 0.0008%) and in gnomAD v4.0.0 (MAF = 0.0002739%). The affected residue is conserved in all vertebrates (Additional file 1: Figure S1) and in silico prediction scores suggest a deleterious effect of the variant (PolyPhen2 = 0.965; SIFT = 0.0; REVEL = 0.418; CADD = 28.8; GERP = 5.19, PhastCons = 1). The mutated Gly11 residue maps to the zinc-finger [23] (Additional file 1: Figure S3). In silico modelling suggests that the zinc atom-coordinated cysteines expose the sidechains of the conserved Pro8, Gly11, Asp30, and Gly32 residues (Fig. 1B right panel, Additional file 1: Figure S3). Further modelling of the entire RNA polymerase complex suggests that Asp30 and Gly32 are in direct contact with the equivalent of RPC1 (POLR3A) and that the side of the zinc-finger bearing Pro8 and Gly11 will be available to bind other molecular entities (Additional file 1: Figure S3).

### *BRF2* variant assessment

**Fig. 2:**
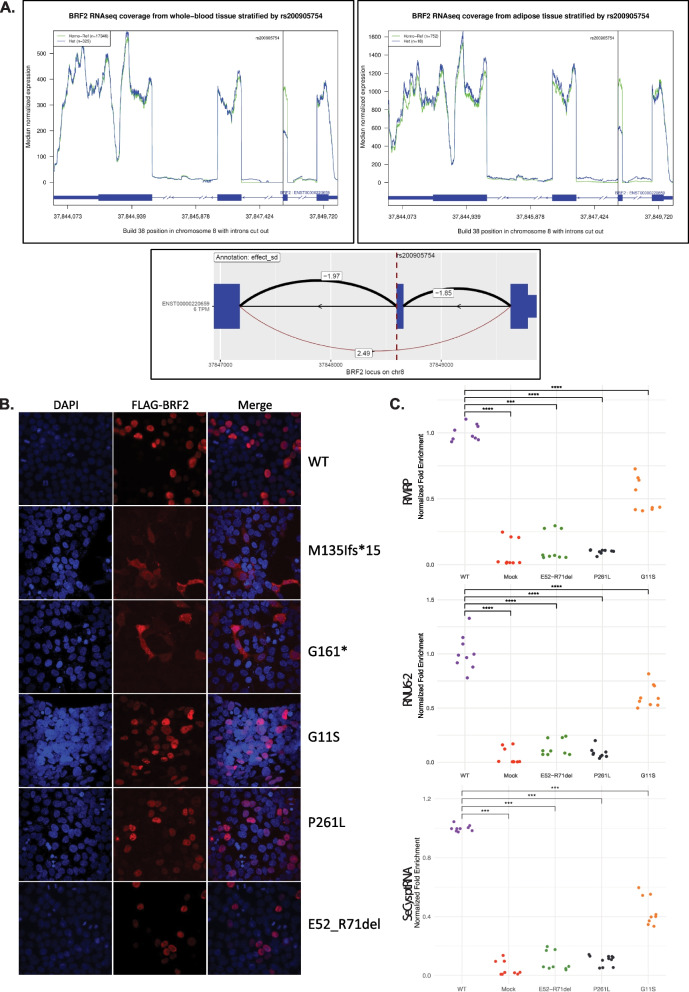
Variants assessments.** A** Effect on RNA expression of the splice donor variant *BRF2*(NM_018310.4):c.214 + 1G > A; p.(Glu52_Arg71del) (a.k.a. rs200905754). The median RNA-sequence coverage is reported for heterozygous (in blue) and noncarriers (in green) in blood (left top panel) and adipose tissue (right top panel). The splicing variant (dashed line in bottom panel) perturbs the correct splicing of exon 2 (black isoform, top cis-sQTL) and induces the skipping of that exon (red isoform: effect = 2.49 SD, *P* = 1.0 × 10^−444^). The splice junction usage quantification was calculated in terms of PSI (white labels). The dark blue squares represent exons of selected BRF2 transcripts which matched exon–intron boundary of the splice junctions. **B** Subcellular localization of FLAG-BRF2. Immunofluorescent staining with DAPI (blue) is shown on the left, FLAG-BRF2 (red) in the middle and the merged signals on the right. **C** ChIP-qPCR normalized fold enrichment analysis of *RMRP, RNU6-2*, and *SeCys ptRNA* loci occupancy by N-terminal FLAG-tagged BRF2. Comparison of FLAG-tagged BRF2 wild-type (WT) and mutants (FLAG-BRF2^E52−R71del^, FLAG-BRF2^G11S^, and FLAG-BRF2.^P261L^) HEK293T transfected cells with mock-treated cells. The statistical significance of pairwise comparisons between conditions was determined using the Wilcoxon rank-sum test, and significant differences are indicated with *p*-value annotations. ****p ≤ 0.0001; ***p ≤ 0.001

We first assessed the effect of the *BRF2* (NM_018310.4):c.214 + 1G > A variant on RNA splicing (sQTL) and mRNA levels (eQTL) in the Icelandic population based on RNA sequencing of blood samples from 17,674 individuals (17,351 non carriers and 323 heterozygous carriers) (Fig. 2A) [78]. Heterozygotes displayed a large increasing effect in quantile-normalized percentage spliced (effect = 2.49 SD, *P* = 1.0 × 10 − 444), corresponding to a 49% increase in exon skipping (Fig. 2A). This effect was also observed in adipose tissue (effect = 2.40 SD, *P* = 1.1 × 10 − 25) (Fig. 2A). These results demonstrate that c.214 + 1G > A results in the skipping of exon 2 and the deletion of twenty amino acids in the encoded protein, i.e., p.(Glu52_Arg71del). The deleted region overlaps the linker that bridges the zinc finger domain with the DNA binding domain of BRF2 and impacts the two first turns of the alpha-helix at the start of the DNA binding domain. Although residues 40 to 65 between the zinc-finger and the DNA binding domain could not be modeled, the residues Ser66 and Ser68 contact the DNA backbone, whereas the Arg67 sidechain reaches the DNA major groove (UniProt: Q9HAW0; Additional file 1: Figure S4). The position of the zinc-binding domain of BRF2 relative to its DNA binding domain can be inferred from the position of the corresponding domains of the *S. cerevisiae* BRF1 crystal structure (pdb entry 6F40). The linker encoded by p.(Glu52_Arg71del) will be too short to maintain both the full length of the first helix of the DNA binding domain and the correct orientation of the zinc-binding domain. We estimate that the distance between the carbon alpha of Val39 (end of zinc-binding domain) and Leu73 (in the first helix of the DNA binding domain, just after the region impacted by the splicing variant deletion) is of 42 Angstroms (Additional file 1: Figure S4). The 15 residues available in the variant protein harboring the deletion will be just enough to cover this distance, provided they adopt an extended conformation (the maximal distance that can be covered by 15 residues adopting a fully extended conformation is 46.2 Angstroms). Consequently, although this variant might still be functional, it will at the very least have an impact on the DNA binding specificity.

We then assessed the effect of the identified *BRF2* variants by transfecting plasmids encoding N-terminal FLAG-tagged BRF2 wild-type (WT) or mutants (FLAG-BRF2^G161*^, FLAG-BRF2^M135Nfs*15^, FLAG-BRF2^G11S^, FLAG-BRF2^P261L^ and FLAG-BRF2^E52_R71del^) in HEK293T cells. We first evaluated expression levels by western blotting (Additional file 1: Figure S5A). Two anti-FLAG reactive bands were observed upon transfection with FLAG-BRF2^WT^, FLAG-BRF2^G11S^, and FLAG-BRF2^P261L^: a weak one at ~ 50 kD and a second main one at ~ 72 kD. While the smaller band corresponds to the expected size of FLAG-tagged BRF2, the cause of the slower migration of the upper band is unknown. Glycosylation, phosphorylation, ubiquitination, and non-separation of protein complexes or aggregates have been shown to perturb SDS-observed molecular weight. Consistent with the deletion of twenty residues, these two bands were observed at a lower size compared to the FLAG-BRF2^WT^ upon transfection with FLAG-BRF2^E52_R71del^. Both bands were not observed in non-transfected cells, showing the specificity of the signal. An anti-FLAG reactive band was seen at the expected weight of ~ 23 kD for the FLAG-BRF2^G161*^ while no signal was detected upon transfection with the FLAG-BRF2^M135Nfs*15^ frameshift variant (Additional file 1: Figure S5A). A weak signal at ~ 20 kD can be observed for the FLAG-BRF2M135Ifs*15 frameshift variant upon loading four times more proteins (Additional file 1: Figure S5B). We then examined the subcellular localization of the mutated BRF2 proteins by immunostaining (Fig. 2B). While FLAG-BRF2^WT^, FLAG-BRF2^G11S^, FLAG-BRF2^P261L^, and FLAG-BRF2^E52_R71del^ showed a nuclear localization (100% (in 22 out of 22 transfected cells), 100% (26/26), 100% (33/33), and 100% (106/106) respectively) in line with the role of BRF2 as a transcription initiation factor, the stop-gain, and frameshift mutant proteins showed cytoplasmic localization (100% (19 out of 19 transfected cells) and 100% (22/22), respectively), suggesting that FLAG-BRF2^G161*^ and FLAG-BRF2^M135Nfs*15^ encode truncated proteins lacking their nuclear localization signal. Our results show that the truncated BRF2 proteins would mislocalize if their corresponding transcripts escape nonsense-mediated decay.

To get a more mechanistic assessment of the impact of the missense and in-frame variants, we then assessed the occupancy of *RMRP*, *RNU6-2*, and *Selenocysteine-tRNA* promoters, three known BRF2-bound loci [24, 79], in HEK293T cells transfected with plasmids encoding N-terminal FLAG-tagged BRF2 wild-type (WT) and mutants (FLAG-BRF2^E52−R71del^, FLAG-BRF2^G11S^, and FLAG-BRF2^P261L^) by ChIP-qPCR (quantitative PCR). We observed a significant occupancy increase when comparing mock- with WT BRF2-transfected cells (*p* < 0.001) demonstrating that the FLAG-tagged BRF2 protein binds the above loci as published [24, 79] (Fig. 2C). Consistent with the 3D models, our results suggest that the Icelandic variant that encodes a protein lacking residues spanning the linker domain, and the P261L and G11S missense variants of families 5 and 6 predicted to affect DNA binding and the N-terminal zinc finger, respectively, have functional impacts as we found that the FLAG-BRF2^E52−R71del^, FLAG-BRF2^P261L^, and FLAG-BRF2^G11S^ isoforms occupy the *RMRP*, *RNU6-2*, and *Selenocysteine-tRNA* loci significantly less than FLAG-BRF2^WT^ (*p* < 0.001) (Fig. 2C). Suggestive of an hypomorph, target occupancy of the FLAG-BRF2^G11S^ protein is significantly increased when compared to mock-transfected cells and both FLAG-BRF2^E52−R71del^ and FLAG-BRF2^P261L^ (*p* < 0.001) (Fig. 2C).

### Zebrafish *brf2* knock-down larvae

**Fig. 3:**
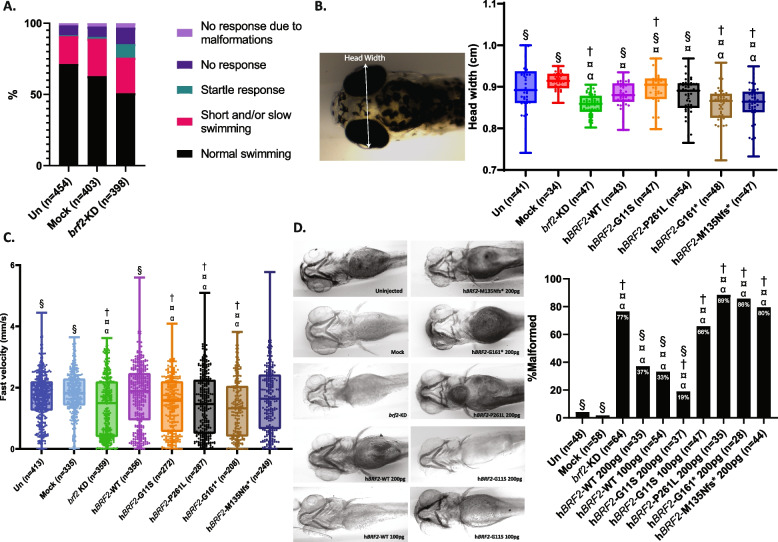
*brf2*-knocked-down zebrafish.** A** Touch-response test showing the percentage of the classified swimming movements upon a tactile stimulus on the tail for uninjected (Un), mock-injected (Mock), *brf2*-knocked down (*brf2*-KD) zebrafish larvae at 3 dpf. The number of tested larvae is indicated in parenthesis. **B** Head width (indicated by white arrows in the left panel) measurements Un-, Mock, brf2*brf2*-KD, and *brf2*-KD co-injected with human mRNA BRF2 wild-type (h*BRF2*-WT), Gly11Ser (h*BRF2*-G11S), Pro261Leu (h*BRF2*-P261L), Gly161* (h*BRF2*-G161*), Met135Asnfs*15 (h*BRF2*-M135Nfs*). The number of tested larvae is indicated in parenthesis. **C** Swimming fast velocity in the dark of Un, Mock, *brf2*-KD, and *brf2*-KD co-injected with human mRNA *BRF2* wild-type (h*BRF2*-WT), Gly11Ser (h*BRF2*-G11S), Pro261Leu (h*BRF2*-P261L), Gly161* (h*BRF2*-G161*), Met135Asnfs*15 (h*BRF2*-M135Nfs*). **D** Alcian blue staining in zebrafish larvae at 5 dpf. On the left, representative ventral pictures of the Alcian blue staining showing jaw malformations of *brf2*-KD co-injected with human RNA *BRF2* Pro261Leu (h*BRF2*-P261L 200 pg), Gly161* (h*BRF2*-G161* 200 pg), Met135Asnfs*15 (h*BRF2*-M135Nfs* 200 pg), and half dose of *BRF2* Gly11Ser RNA (h*BRF2*-G11S 100 pg) compared with illustrative pictures of the normal jaw structure observed in Un, Mock, and *brf2*-KD co-injected with human *BRF2* wildtype at normal (h*BRF2*-WT 200 pg) and half dosage (h*BRF2*-WT 100 pg) and Gly11Ser (h*BRF2*-G11S 200 pg). The fraction of the observed deformed jaw structure is presented in the right panel as a percentage. *** *P* < 0.001; ** *P* < 0.005; ns = not significant; § significant vs brf2- KD (*P* < 0.05); ¤ significant vs Mock (*P* < 0.05); † significant vs h BRF2 -WT (*P* < 0.05); α significant vs Un (*P* < 0.05)

To gain insights into the function of *BRF2*, we ablated its one-to-one ortholog in zebrafish, *brf2*, by CRISPR-Cas9 genome editing. To ensure maximum efficiency in engineering F0 knock-down (KD) larvae, we used three different single-guide RNA (sgRNA) targeting exons 3, 4, and 5 of *brf2*, with efficiencies of 90, 80, and 100%, respectively (Additional file 1: Figure S6A). Importantly, all assessed larvae were altered by at least two of our sgRNAs (Additional file 1: Figure S6A). At 3 days post fertilization (dpf), we assessed the escape response upon a tactile stimulus of larvae by touch-response test. We observed an increased fraction of *brf2*-KD larvae with a startle response (uninjected = 0.9%; mock-injected = 1.2%; *brf2*-KD = 9.6%) and with an inefficient escape response (uninjected = 6.9%; mock-injected = 7.2%; *brf2*-KD = 11.8%) compared to un- and mock-injected larvae (Fig. 3A). The inefficient escape response was not driven by malformed larvae as they were considered in a separate category (No response due to malformations: uninjected = 1.5%; mock-injected = 2.5%; *brf2*-KD = 3.0%). Five dpf *brf2*-KD larvae showed a significant reduction in swimming velocity (Fig. 3C). This impaired response was not due to malformed larvae as the 6.5% (26 out of 398) of 5 dpf malformed larvae were excluded from this experiment. They presented with a specific phenotype with skeletal and head malformations (Additional file 1: Figure S6A). The reduction in swimming velocity was not due to changes in the response to light because no significant differences in the visual motor response of *brf2*-KD, uninjected and mock-injected fish larvae were observed during light–dark transitions (Additional file 1: Figure S6C). Quantification showed that 5 dpf *brf2*-KD larvae presented with a significantly smaller head width compared to uninjected and mock-injected larvae (Kruskal–Wallis test: *brf2*-KD vs uninjected: *P* < 0.0001; *brf2*-KD vs mock-injected *P* < 0.0001; Fig. 3B). Likewise, cartilage staining of 5 dpf zebrafish larvae revealed craniofacial anomalies in *brf2*-KD larvae, mainly in the ceratohyal cartilage (fraction of malformed: uninjected = 4.2%; mock-injected = 1.7%; *brf2*-KD = 77%; Fig. 3D).

We then co-injected human *BRF2* WT RNA with the CRISPR-Cas9 guides to check if it was able to rescue the observed phenotype, as orthologues proteins usually retain the same function [80]. Of note, the BRF2 protein alignment showed that compared to other vertebrates in some fish species (e.g., zebrafish, catfish, eel, and piranha) the position corresponding to human Pro261 (a threonine in zebrafish) is preceded by a longer loop and needs to be considered as a unit connecting helices (Additional file 1: Figure S1). In human, Pro261 forms the boundary between a loop and a helix and the DLPY residues of this loop preceding Pro261 have no close corresponding residues in zebrafish as demonstrated by their high root mean square deviation (rmsd) (Additional file 1: Figure S1). Despite this difference, co-injection of human *BRF2* WT RNA with the CRISPR-Cas9 guides complemented the hypolocomotion, the head width, and craniofacial phenotype (Fig. 3B–D), demonstrating that ablation of *brf2* is causative of the zebrafish phenotypes and allowing us to further assess the *BRF2* variants. We included the two truncation variants of families 4 and 5 in these experiments as negative controls. As expected, the two truncating variants were not able to rescue any of the assessed phenotypes, confirming their deleteriousness (Fig. 3B–D). The two missense variants complemented the smaller head width but not the hypolocomotion (Fig. 3B,C). Co-injection of *BRF2* Gly11Ser RNA could avert the jaw malformations contrary to the Pro261Leu RNA that did not complement this phenotype (Fig. 3D). We repeated the same experiment co-injecting only half of the *BRF2*-WT and Gly11Ser mutant RNA quantity (100 pg). Whereas halving the human *BRF2*-WT RNA still complemented the craniofacial phenotype, a similar reduction of the Gly11Ser RNA failed to rescue this phenotype (Fig. 3D). Our results suggest that Gly11Ser and Pro261Leu, which rescue two (one in a dose dependent fashion) and one of the three assessed phenotypes, respectively, act as hypomorphs.

## Discussion

We identified biallelic variants in *BRF2* in seven individuals from three unrelated families and found no homozygous carriers of an Icelandic *BRF2* founder splicing variants within 29 offspring of pairs of heterozygote parents. Our 3D protein modelling, transcriptome profiling, and in vitro and in vivo assays support the causativeness of the reported variants. Consistent with the association between *BRF2* and a severe autosomal recessive disease, neither homozygous nor compound heterozygous *BRF2* variants classified as “weak missense variant or worse,” i.e., with a REVEL score ≥ 0.644, were identified in gnomAD v2.1.1 [19].

Biallelic pathogenic variants in genes encoding for RNA polymerase III subunits have been associated with a wide clinical spectrum. For instance, pathogenic variants in two subunits of RNA polymerase III, *POLR1C* and *POLR1D*, were previously linked to Treacher-Collins syndrome (MIM#248390, #613717) [35, 81]. These two, as well as other RNA polymerase III subunits, were also linked to hypomyelination leukodystrophies [36, 82], while *BRF1*, the *BRF2* paralog, was associated with CFDS [37–41]. The identified pathogenic variants were suggested to act by decreasing the polymerase-III function and perturb the transcription of its targets [34, 82–84]. The mapping of several variants showed that they might impair the abundance, assembly, and/or activity of the RNA-polymerase III complex possibly explaining the phenotypic heterogeneity of affected individuals [85, 86].

Similarly, the BRF2 affected individuals did not present with a distinctive clinical presentation but rather with overlapping yet different phenotypes ranging from perinatal death to Treacher-Collins and craniosynostosis with radial defects and immunodeficiency or global developmental delay, hearing, and vision impairment. This is consistent with the view that novel Mendelian disorders are likely to be less penetrant and show a more variable expressivity, as postulated in [87]. These variable phenotypes might be caused by different molecular mechanisms of action of discrete pairs of variants, i.e., homozygous LoF, homozygous in frame deletion, compound heterozygous between a truncating and a missense variant in proximity to the DNA binding domain, and a homozygous missense variant in the N-terminal zinc finger.

We reported on a likely Icelandic founder variant that induces the skipping of *BRF2* exon 2 resulting in an in-frame deletion of 20 codons (Fig. 2A). This shortens the linker that bridges the zinc-binding and the DNA-binding domains of BRF2 (Additional file 1: Figure S4) and results in a significant decrease in BRF2 target loci occupancy (Fig. 2C). While this variant is present in Iceland with a MAF of 0.83%, no homozygous individuals are observed in population databases. Such a deficit of pLoF variants homozygosity was observed in genes that cause embryonic lethality and/or are essential for cell line viability [16, 17]. Autosomal recessive genes were similarly found to lack homozygous pLoF [18, 20]. Correspondingly, we report that among the seventeen offspring of three Icelandic pair of heterozygous parents, five were either stillborn or died soon after birth, while all surviving siblings are heterozygous (Fig. 1A). In addition, *BRF2* was reported to be essential in multiple human cell lines (HAP1, HCT, HeLa, GBM, RPE1, DLD1, K562, jiyoye, raji) [88–90]. The subcellular localization and our in vivo rescue assays suggest that the two truncating variants of families 4 and 5 are also functional nulls, pinpointing that in most families described here the most likely disease mechanism is the loss of BRF2 function. Silencing of *BRF2* in lung cancer cells inhibits cell proliferation and migration, while promoting cell apoptosis [91]. BRF2 and its paralogue BRF1 have mutually exclusive target regions [92] and among SNAPc-dependent promoters about a dozen of snRNA loci are exclusively occupied by BRF2 and RNA Polymerase III and cannot be bound by GTF2B and RNA Polymerase II [79]. Specific BRF2-dependent transcripts include the selenocysteine tRNA, the spliceosomal U6 small nuclear RNA (snRNA), the RNA component of the tRNA processing enzyme RNAse P, and the mitochondrial RNA-processing endoribonuclease (RMRP), which are involved in essential cellular function [79, 92, 93].

The p.(Pro261Leu) variant identified in family 5 is likely functionally similar to the *BRF1* p.(Thr259Met) variant identified in a CFDS family (Additional file 1: Figure S2B). Since the proline residue is substituted by a larger leucine residue carrying a hydrophobic side chain it could interfere with DNA binding (Fig. 1A). Consistent with this hypothesis, we found that this variant binds to the *RMRP*, *RNU6-2*, and *Selenocysteine-tRNA* loci less than its WT counterpart (Fig. 2C). In zebrafish, it failed to rescue the hypolocomotion and the alteration of the jaw cartilage (Fig. 3C,D), while it complemented the smaller head (Fig. 3B) and bound target loci (Fig. 2C). Similarly, the *BRF1* Thr259Met paralogous variant scored as hypomorphic in similar rescue experiments of morpholino-ablated *brf1a* and *brf1b* zebrafish, including a partial rescue of the head width [37]. While it was able to rescue the growth of yeast lacking the orthologous *BRF1*, it showed impaired RNA polymerase III-dependent transcriptional activity. Correspondingly, the encoded mutant *BRF1* protein showed decreased promoter occupancy at tRNA loci and the U6 snRNA promoter [37], in line with the reduced occupancy of BRF2^P261L^ at the *RMRP*, *RNU6-2*, and *Selenocysteine-tRNA* promoters (Fig. 2C). The *BRF2* missense variant p.(Gly11Ser) identified in the sixth family maps to the N-terminal Zinc-ribbon domain. It modifies a segment that is locked in a rigid conformation by the four conserved Cys7, Cys10, Cys28, and Cys 31 residues involved in the coordination of the Zinc atom to expose at the surface the adjacent conserved residues Pro8, Gly11, Asp30, and Gly32 that can interact with other polymerase III subunits via their sidechains (Fig. 1B right panel, Additional file 1: Figure S3). In vitro assays showed that although ablation of this domain did not prevent binding to a TBP/TATA box complex, recruitment of BDP1, or interaction with SNAPc, it was essential for U6 transcription [23]. In line with studies on *BRF1* [94, 95], it was hypothesized that the Zinc-ribbon of BRF2 might be likewise required for promoter opening [23]. In the current study, we coincidingly showed that the BRF2^G11S^ mutant RNA was unable to rescue hypo-locomotion, yet it was still binding to target loci (Fig. 2C) and was proficient in restoring the craniofacial anomalies. However, the cartilage structure of the jaw could not be restored when its amount was reduced (Fig. 3D), supporting the hypothesis that it might act as a hypomorph. Consistent with the observed reduced deleteriousness of this missense variant in the Zinc-ribbon domain, (i) the decrease in target loci occupancy is less striking than that observed for the other assessed variants (Fig. 2C) and (ii) the affected siblings of the sixth family present with a milder phenotype that lacks perinatal death and overt craniofacial anomalies when compared to the other affected individuals presented in this study.

## Conclusions

We provided evidence for an association between biallelic *BRF2* variants and craniofacial anomalies and early death. Our results further support the postulate that an intact RNA polymerase III complex is required for normal development.

## Supplementary Information

Additional file 1: Table S1. Identified homozygous by WES variants in individual II:1, 2 and 3 of family 6Table S2. Table S2. sgRNAs sequences and their corresponding primer pairs. Figure S1. Protein alignment of BRF2 in vertebrates. Figure S2. 3D model of the BRF2-Pro261 and BRFs cyclin repeats bound to DNA. Figure S3: The relative position of BRF2 to RNA polymerase III. Figure S4. 3D model of the deleted inframe region p.(Glu52-Arg71del). Figure S5: Western blot of FLAG-BRF2- transfected HEK293T cells. Figure S6. Characterization of brf2-knock down (KD) zebrafish.

## Data Availability

No datasets were generated or analysed during the current study.
