# Supplementary material for: Bi-allelic variants in BRF2 are associated with perinatal death and craniofacial anomalies
Source: Genome Med. 2025 Apr 14;17:38. doi: 10.1186/s13073-025-01463-3 (PMC11995667; doi:10.1186/s13073-025-01463-3)
Supplement: Supplementary file 1 — Additional file 1: Table S1. Identified homozygous by WES variants in individual II:1, 2 and 3 of family 6Table S2. Table S2. sgRNAs sequences and their corresponding primer pairs. Figure S1. Protein alignment of BRF2 in vertebrates. Figure S2. 3D model of the BRF2-Pro261 and BRFs cyclin repeats bound to DNA. Figure S3: The relative position of BRF2 to RNA polymerase III. Figure S4. 3D model of the deleted inframe region p.(Glu52-Arg71del). Figure S5: Western blot of FLAG-BRF2- transfected HEK293T cells. Figure S6. Characterization of brf2-knock down (KD) zebrafish. [file 13073_2025_1463_MOESM1_ESM.pdf]

Table S1

Chrom	Start	Ref	Alt	Genotype	HGVS	Gene_symbol	PanelAPP_ID	Variant c.	Variant p.	Aa_change	Impact	CADD_score_scaled	GERP_score	Polyphen_pred	Polyphen_score	Sift_pred	Sift_score	
chr8	37707271	C	T	Homozygous in all 4 affected siblings	NM_018310.4:c.31G>A; p.(Gly11Ser)	*BRF2*	no	ENST00000220659.6:c.31C>A	ENSP00000220659.6:p.Gly11Ser	G/S	missense_variant	28.8	5.19	probably_damaging	0.965	deleterious	0.0	
chr7	73638503	T	C	Homozygous in all 4 affected siblings	ENST00000460943.1:c.604T>C; p.(Trp202Arg)	*LAT2*	no	ENST00000398475.1:c.604G>C	ENSP00000381492.1:p.Trp202Arg	W/R	missense_variant	26.9	4.09	probably_damaging	0.996	deleterious	0.0	Linker for activation of T-cells family member 2
chr8	42176166	G	A	Homozygous in all 4 affected siblings	NM_001556.3:c.1337G>A; p.(Arg446Gln)	*IKBKB*	no	ENST00000416505.2:c.1160T>A	ENSP00000404920.2:p.Arg387Gln	R/Q	missense_variant	23.3	4.71	benign	0.019	tolerated	0.12	Associated with immunodeficiency 15A (#MIM:618204) and 15B (#MIM:615592)
chr8	38854108	G	A	Homozygous in individuals 1, 2,3	ENST00000520152.1: c.-191+4C>T	*TM2D2*	no	ENST00000520152.1: c.-191+4C>T	\	\	splice_region_variant	8.2	0.72	\	\	\	\	PhastCons=0,055; phyloP=0,248
chr7	76028066	G	A	Homozygous in individuals 1, 2,3	NM_080744.2:c.524C>T; p.(Ala175Val)	*SRCRB4D*	no	ENST00000275560.3:c.524G>T	ENSP00000275560.3:p.Ala175Val	A/V	missense_variant	3.16	0.61	benign	0.003	tolerated	0.2	Low prediction scores
chr8	30703871	ATTG	A	Homozygous in all 4 affected siblings	NM_001350162.2:c.3809_3811del; p.(Thr1270del)	*TEX15*	no	ENST00000256246.2:c.2662_2664delGCA	ENSP00000256246.2:p.Thr888del	TI/I	inframe_deletion		0.24	\	\	\	\	Associated with spermatogenic failure (#MIM:617960)

Table S1: Identified homozygous by WES variants in individual II:1, 2 and 3 of family 6

Table S2

Exon	sgRNA	Forward primer	Reverse primer
3	CCCTGTCGAAATCTAGTCTC**TGG**	CACAGCAGAGGTAAAACACTCA	ATGAACTGAACCTGCATCCG
4	GGTAATTGTCAGCATCCAGC**AGG**	ATCGGCGAATCTCTTTGAGC	ACCTACCCTGACCCGATCTA
5	CTTCTTAGCTGCGGTTTATG**TGG**	TGATCCATGCACTAATTTGAGA	GAATGAATGCTTTCCGGATGC

Table S2: sgRNAs sequences and their corresponding primer pairs

Fig. S1

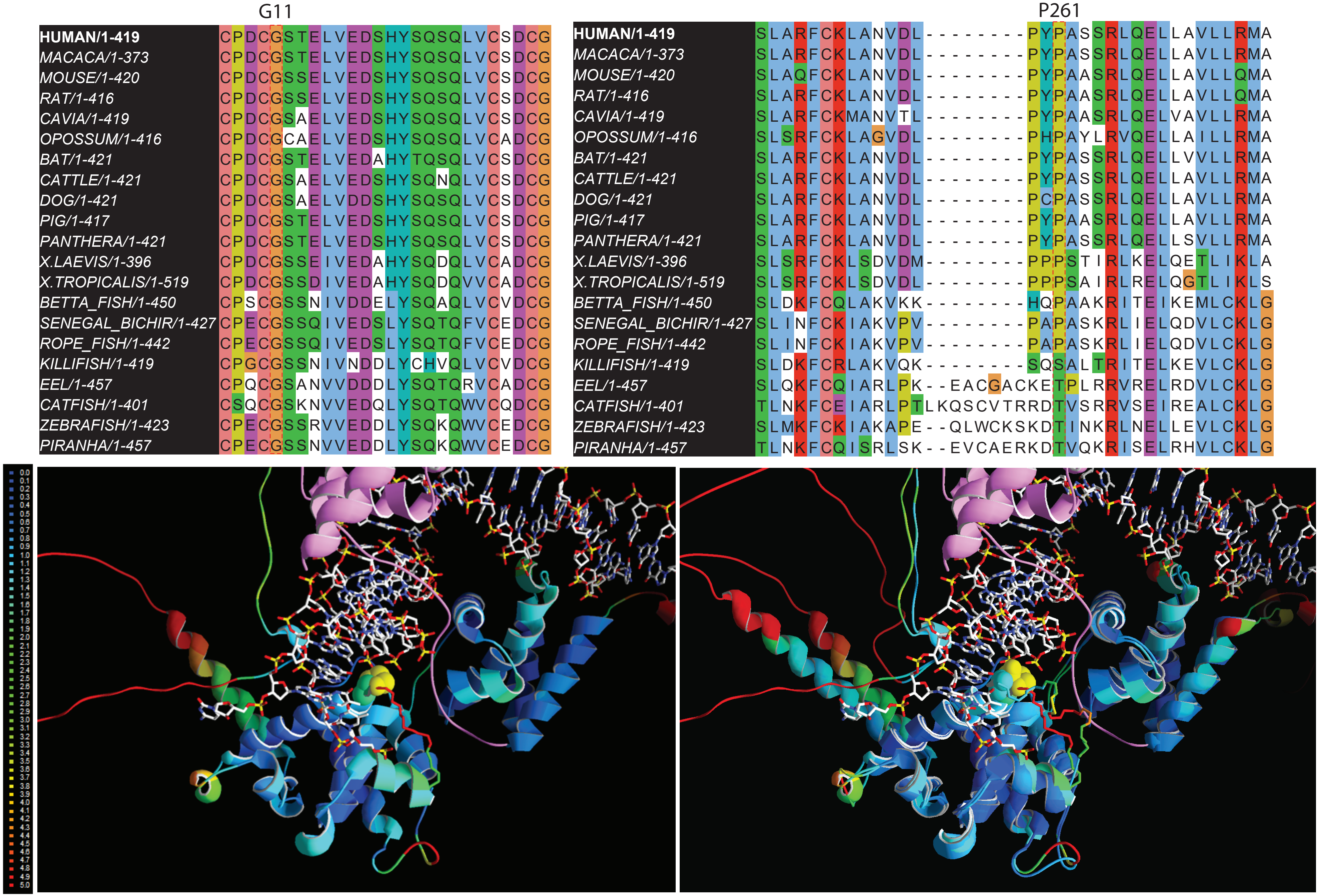

Figure S1: Protein alignment of BRF2 in vertebrates. On the top: Clustal-O alignment using Jalview with protein sequence retrieved from UniProt. Human Gly11 and Pro261 corresponding residues are highlighted with a red rectangle. On the bottom: on the left, overview of BRF2 binding to DNA. DNA and BDP1 (pink) are taken from PDB entry 5N9G. TBP is hidden for clarity. P261 sidechain, which is close to the DNA backbone is shown in yellow spacefill at the center of the image. BRF2 ribbon is colored by rmsd (color scale to the left) to the alphafold Channel catfish model, which has the longest loop. On the right, 3D superposition of Zebrafish model and human structure.

Fig. S2

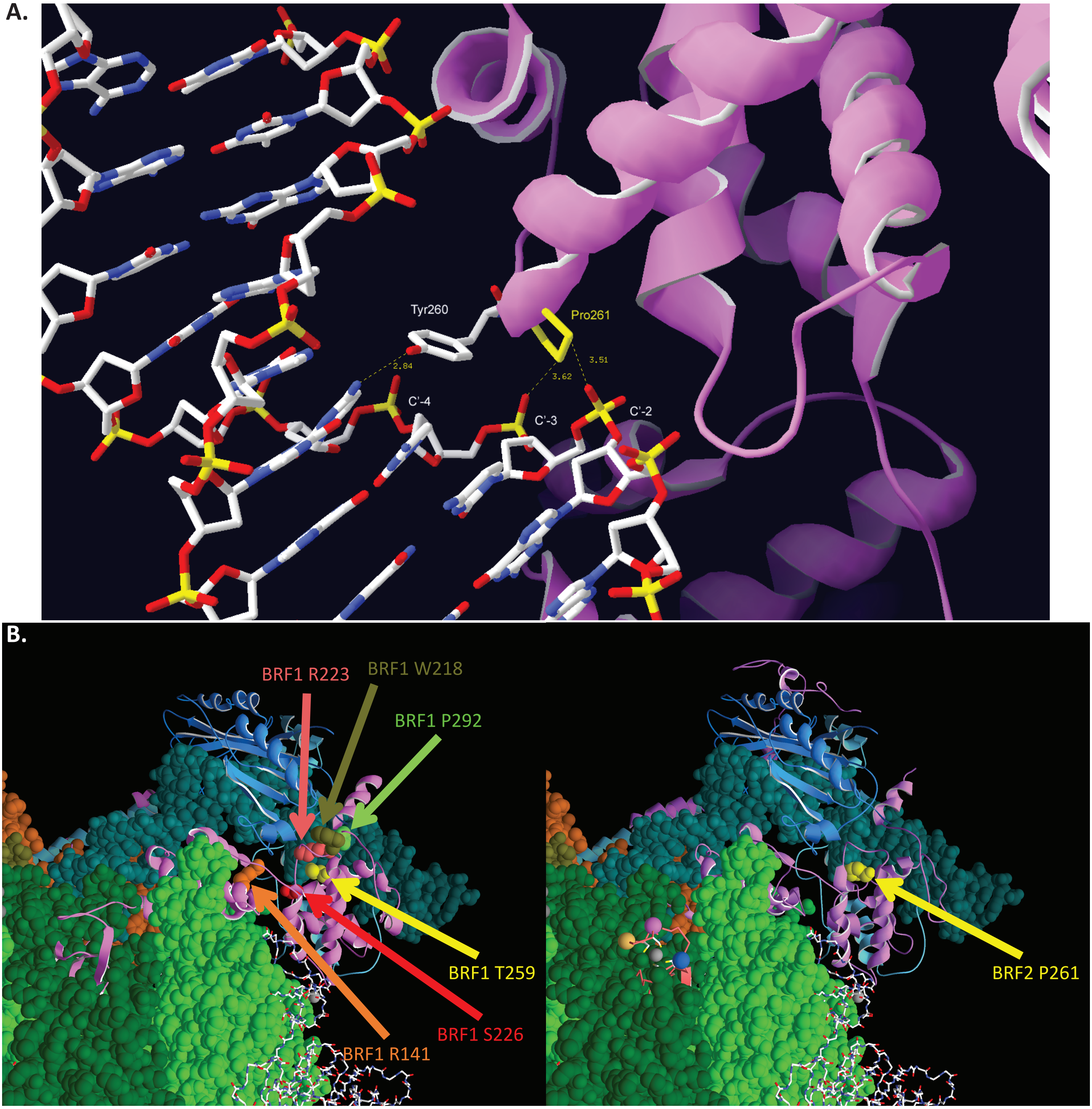

Figure S2: 3D model of the BRF2-Pro261 and BRFs cyclin repeats bound to DNA. A. Closeup view of DNA contact made by BRF2 Tyr260 and Pro261 showing that Tyr260 is in in contact with the template strand nucleotide C'-4 of the TATA box while Pro261 is in contact with the template strand DNA backbone at positions -2 and -3 relative to the TATA (pdb: 4ROC). B. 3D model of BRF (pink ribbon), TBP (sky-blue ribbon), BDP1 (light-blue ribbon), and other RNA polymerase III subunits (TBP chain U shown in sky-blue, BDP1 chain W in light-blue, RPC1 chain A in dark green, RPC2 chain B in light green, RPC3 chain O in kaki, RPC6 chain P in orange, RPC7 chain Q in yellow, RPC8 chain G in grey, RPC9 chain D in brown) bound to DNA (teal). On the left, BRF1 Alphafold model AF-Q92994-F1-model_v2.pdb was superposed on chain V of pdb entry 6F40 retaining the best superposition of the "Explore Fragments Alternate Fits" option of SwissPdbViewer. The region spanning Met1 to Arg302 was rendered as a pink ribbon and the residues mutated in CFDS affected individuals Arg141 (tangerine), Trp218 (asparagus), Arg223 (salmon), Ser226 (maraschio), Thr259 (yellow), and Pro292 (green) are shown in space fill and labelled. All CFDS residues map to the cyclin repeats and are close to the DNA. On the right, BRF1 is replaced by BRF2 (lavender ribbon) and the BRF2-Pro261 is highlighted, in yellow space and labelled. Note that the 3D localization of BRF1-Thr259 (left panel) structurally overlaps that of BRF2-Pro261 (right panel), depicted on the right.

Fig. S3

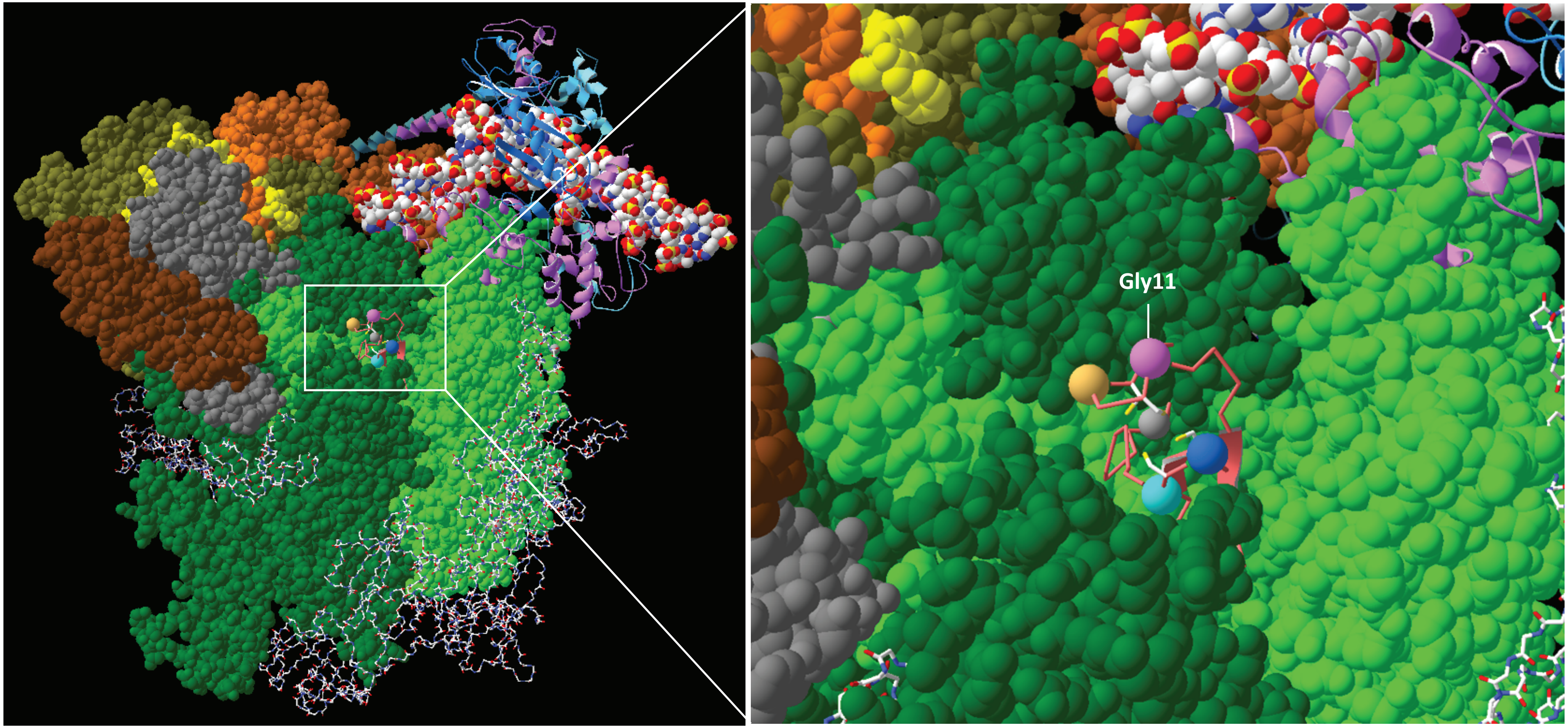

Figure S3: The relative position of BRF2 to RNA polymerase III. The relative position of BRF2 (lavender) to RNA polymerase III (see legend of Additional file 4: Figure S2 for color codes) showing that the N-terminal zinc-ribbon domain, bearing the Gly11 (showed in purple and mutated in family 3), Asp30 (dark-blue residue), Gly32 (light-blue residue) and Pro8 (gold residue) surrounding the zinc-atom (in grey), appears in the middle of the RNA polymerase III complex. A zoom-in view highlighting the solvent-exposed position of Gly11 in the zinc-finger domain is shown on the right.

Fig. S4

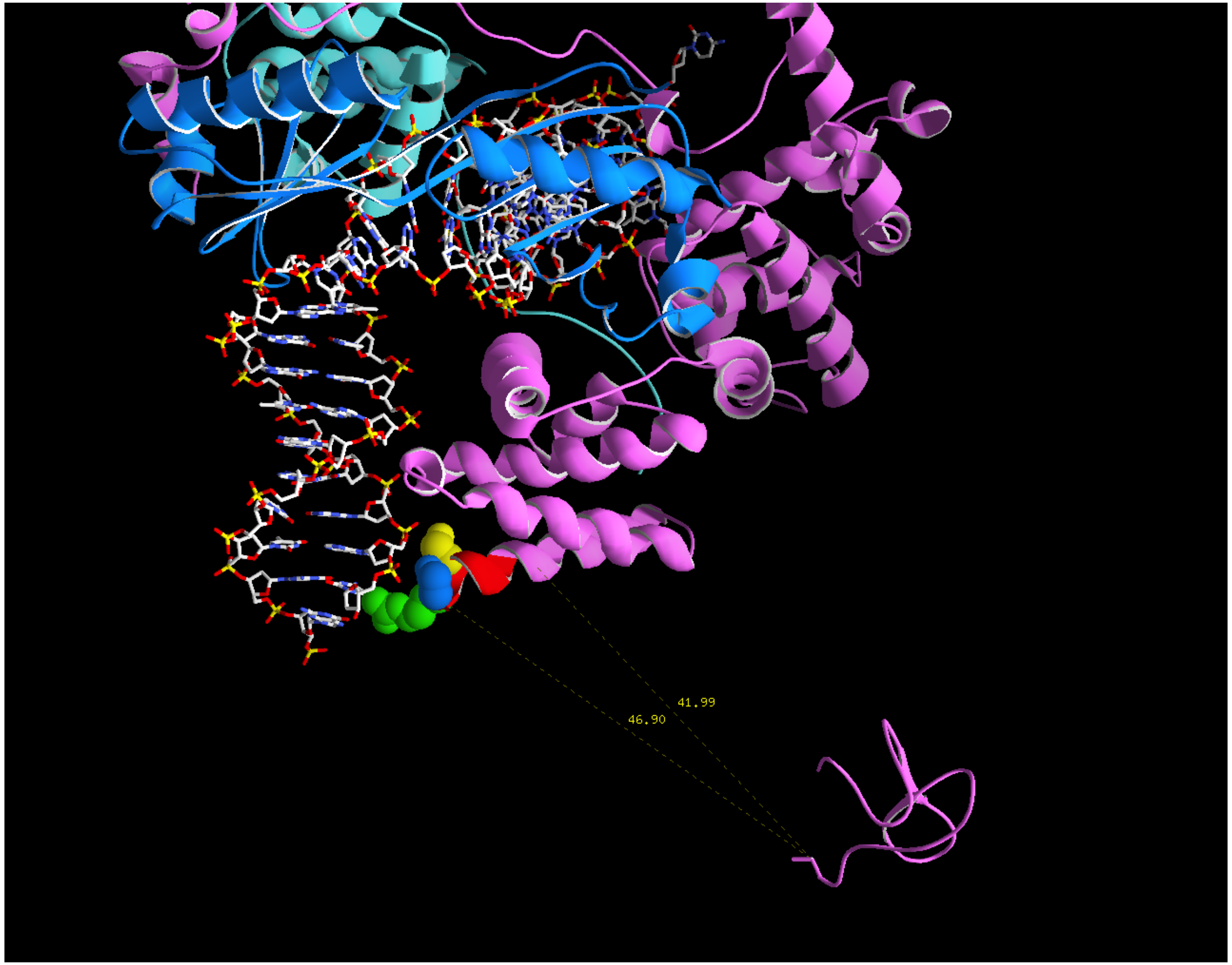

Figure S4: 3D model of the deleted inframe region p.(Glu52-Arg71del). Overview of the topology of BRF2 based on PDB entry 5N9G (BRF2_HUMAN chainA lavender; TBP_HUMAN chain B blue; BDP1_HUMAN: chainC lightblue). The position of the N-terminal domain (bottom right) is inferred from the position of the N-terminal domain of S. cerevisiae BRF1 in complex with RNA Polymerase III open complex (PDB entry 6F40). Sidechains of Ser66, Arg67 and Ser68, all in contact with the DNA, are shown in blue, green and yellow, respectively. They are in a region absent in the splice variant (red ribbon).

Fig. S5

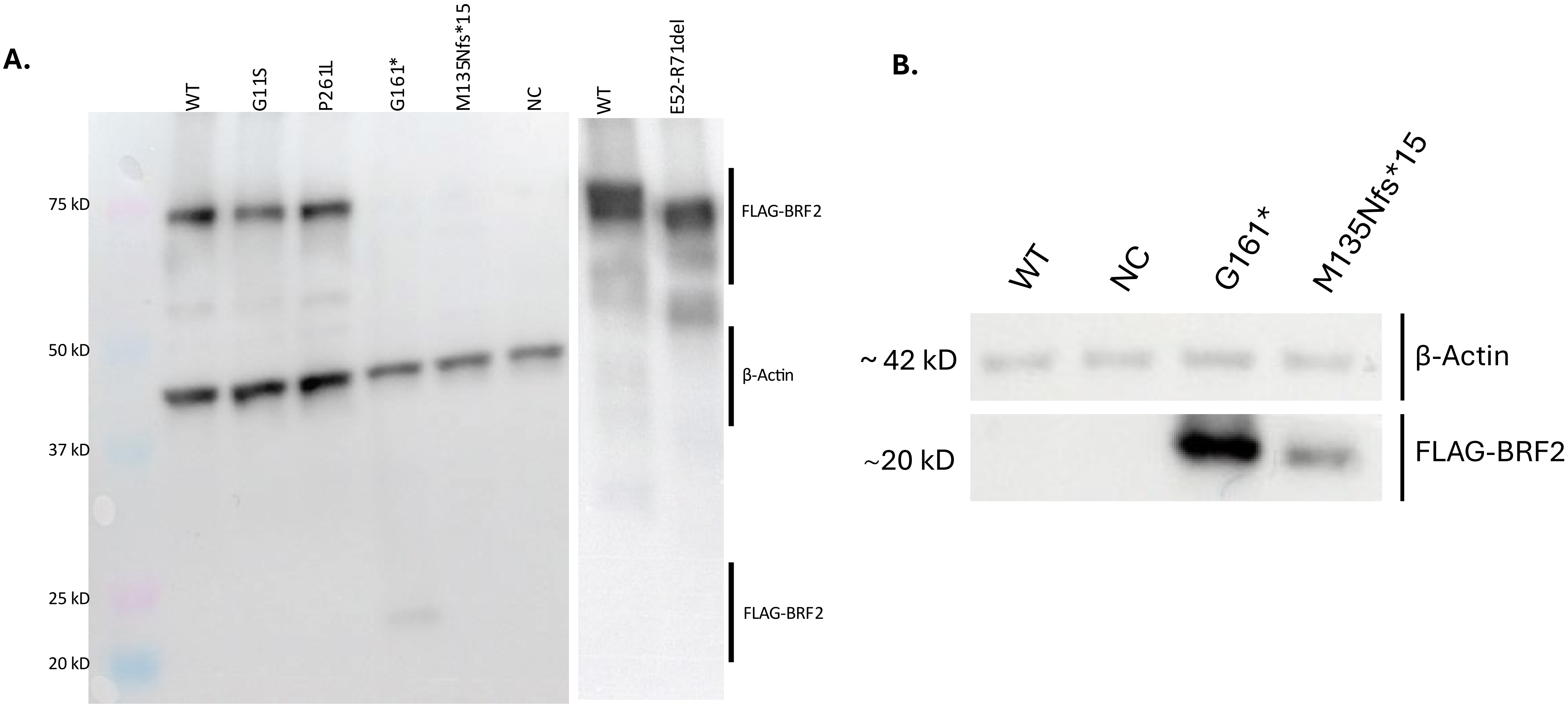

Figure S5: Western blot of FLAG-BRF2- transfected HEK293T cells. A. Not transfected cells (NC) were used as negative control and expression level of β-actin as loading control. Molecular sizes are indicated on the left. **B.** Western blot upon loading four times more wild-type and truncated proteins.

Fig. S6

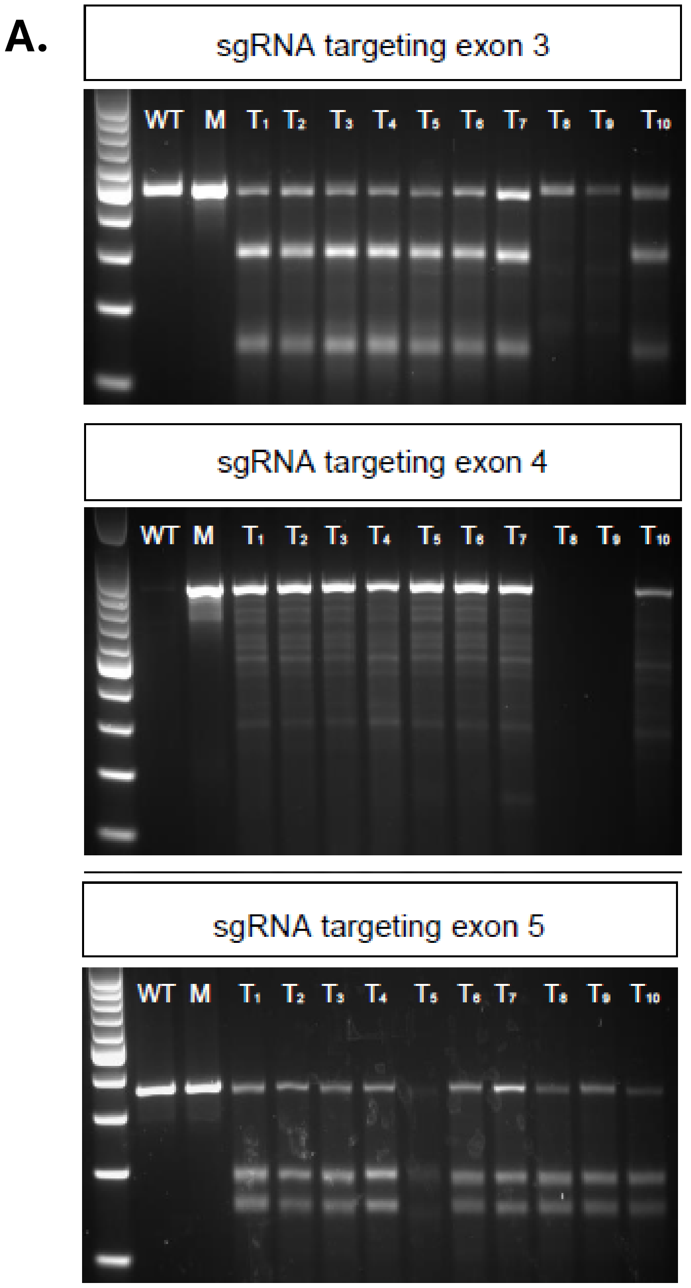

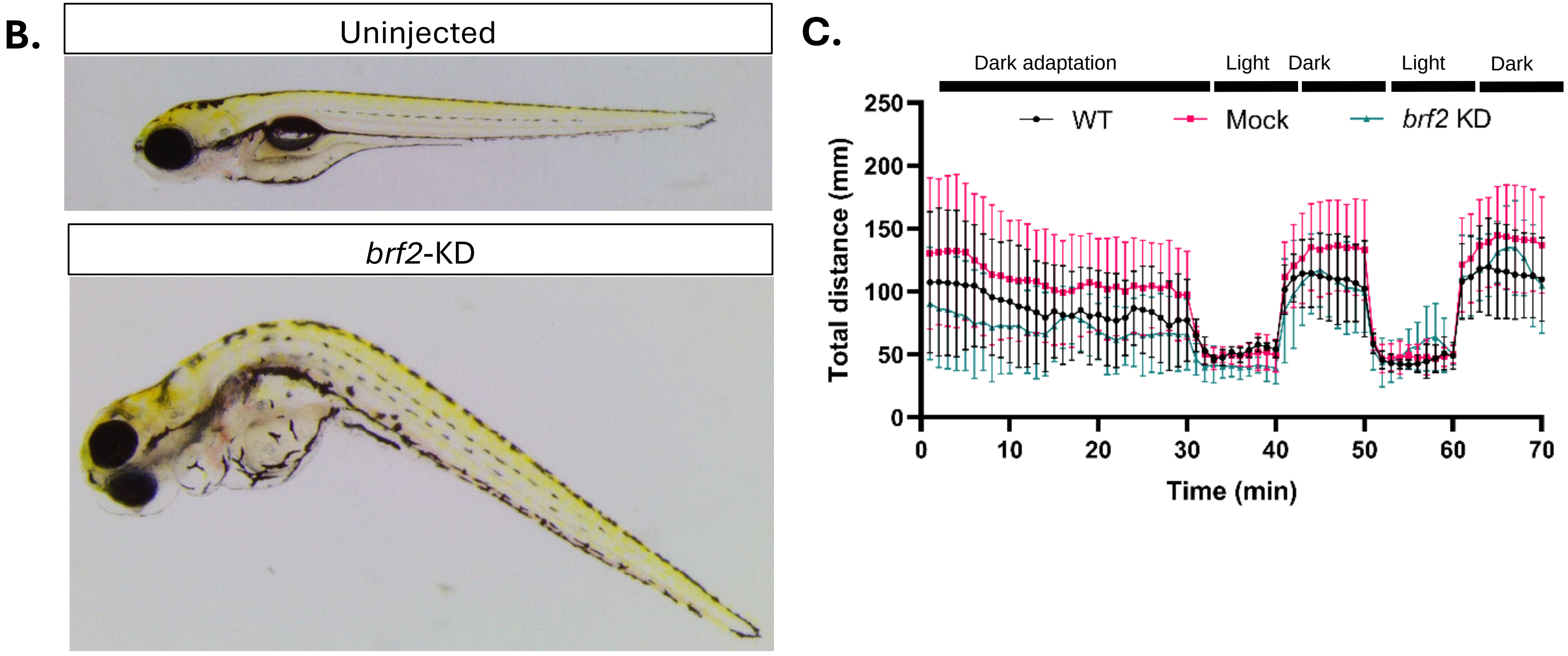

Figure S6: Characterization of brf2-knock down (KD) zebrafish. A. Characterization of CRISPR-Cas9 targeting efficiency of each sgRNA using the T7 endonuclease I on the same uninjected (WT), mock (M) and ten CRISPR/Cas9-treated (T) larvae. Samples with multiple bands indicate a successful CRISPR-Cas9 cleavage. B. Illustrative picture of the observed specific phenotype in ~6.5% of brf2-KD zebrafish larvae with skeletal and head malformation. C. The visual motor response was analyzed at 5 dpf by calculating the total covered distance in an adaptation phase, followed by alternating light conditions. Mean ± SD are indicated. Sample size from three independent experiments: WT, n = 138; Mock-injected, n = 162; brf2 KD, n = 180.
